# A rare case of a phyllodes tumour of the breast converting to a fibrosarcoma with successful treatment

**DOI:** 10.3332/ecancer.2012.247

**Published:** 2012-03-13

**Authors:** NK Pant, A Singh, D Kumar, H Pandey

**Affiliations:** 1 Department of Radiotherapy and Clinical Oncology, Government Medical College, Haldwani, Uttarakhand, India; 2 Department of Radiotherapy and Clinical oncology, V.C.S.G.G.M.S.& R.I., Srinagar Garhwal, Uttarakhand, India; 3 Department of Radiotherapy, Delhi State Cancer Institute, New Delhi, India; 4 Department of Pathology, Government Medical College, Haldwani, Uttarakhand, India

## Abstract

A phyllodes tumour of the breast converting to fibrosarcoma of the breast is a rare entity. Prognosis of fibrosarcoma of the breast is poor and the role of various treatment modalities is not clearly defined due to the rarity of the disease. One such case, which was treated successfully with a combination of surgery, radiotherapy and chemotherapy, is presented here.

## Background

Phyllodes tumours of the breast are commonly classified as benign tumours and rarely as borderline or malignant. Cases have been reported where originally benign tumours developed malignant features with recurrences [1]. Primary sarcomas of the breast are extremely rare and account for less than 0.1% of all malignant tumours of the breast [2], of which the conversion of a phyllodes tumour to sarcoma of the breast is even rarer and only one such case has ever been reported [3]. Prognosis of these rare tumours is poor and treatment modality is not clearly defined. The rarity of this tumour and its successful treatment prompts us to report this case.

## Case history

**Figure 1:**
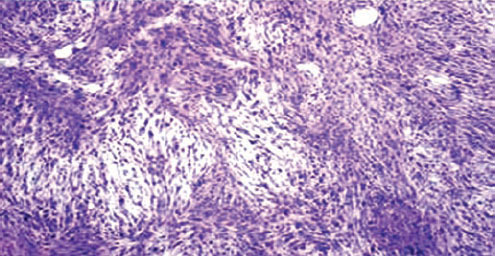
Microphotograph of histopathological examination of third operated tumour showing uniform cellular tumour with spindle shaped cells of varying degrees of pleomorphism, vesicular eccentric nuclei with coarse chromatin and few mitotic figures; collagenous fibres arranged in intertwining whorled bundles; few areas of chondroid with no osteoid differentiation. The fat surrounding the tumour shows strands of normal breast tissue.

**Figure 2:**
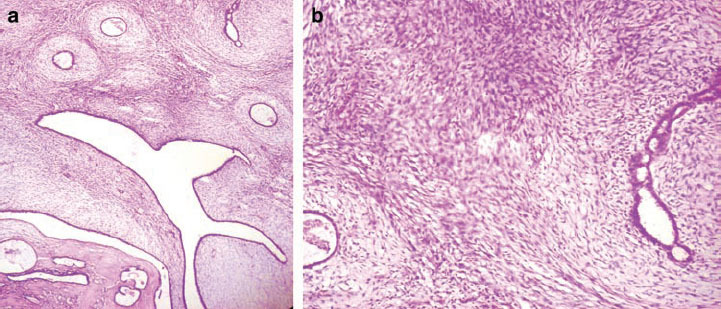
(a) Microphotograph of first surgical specimen: magnification 100X showing moderate stromal hypercellularity with mild nuclear atypia/pleomophism of the spindle cells and myxomatous stromal overgrowth. Focal mildly atypical epithelial hyperplasia was also noted, suggestive of phyllodes tumour (Borderline type). (b) Microphotograph of first surgical specimen: magnification 400X.

**Figure 3:**
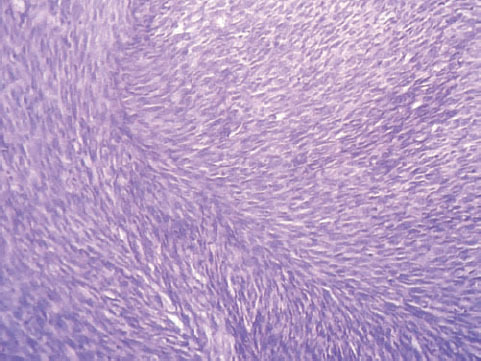
Photomicrograph of the histopathological slide after the final surgery: uniform spindle cells showing little variation in size and shape and a distinct fascicular arrangement. 400X magnification; H&E stain.

A 35-year-old female presented at our institution six weeks after a third surgery for recurrent tumours in the left breast over a period of six years. She complained of mild pain at the operative site. On examination, a healthy scar was present with mild induration. No palpable lymph nodes were present. No abnormality was detected abdominally or in the right breast and the axilla. A complete systemic examination of the patient did not show any other abnormality. The histopathological diagnosis from the last operative specimen was of fibrosarcoma of the breast (Figure 1) while the histopathological examination of both the earlier operative specimens were in favour of a phyllodes tumour (Figure 2a, 2b). A review of all the slides was carried out. The histopathological examination (HPE) analysis of the specimen slide after the second surgery showed a tumour with stromal hypercellularity and the presence of benign glandular elements. The features of the tumour cells were largely those of fibroblasts, accompanied by focal myoid differentiation. Focal fibromyxoid areas were also encountered. Although the stroma was more cellular, the tumour cells were bland-looking, did not show any pleomorphism and mitoses were infrequent. The features were suggestive of a borderline phyllodes tumour. The HPE analysis of the recurrence slide i.e. after the last surgery, clearly displayed a sarcomatous change, characterised by stromal overgrowth and hypercellularity, nuclear atypia and increased mitotic count. The cells were spindle-shaped and varied little in size and shape, had scanty cytoplasm with indistinct cell borders, and were separated by interwoven collagen fibres arranged in a parallel fashion, favouring the diagnosis of fibrosarcoma (Figure-3).

The X-rays of the skeleton and lungs were normal. An abdominal ultrasound was normal. Her left ventricular ejection fraction was 60 percent. Biochemical investigations did not reveal any significant abnormality. A computed tomography scan of the chest and axilla was suggestive of oedema or inflammation of the operative site. Taking into consideration that a lumpectomy rather than a mastectomy was performed with no comment on the status of the histopathology of the margins, the decreasing time interval between each recurrence and the potential histopathological conversion to a malignant phenotype, it was decided, in a multi-disciplinary meeting, that the patient should be given chemotherapy in case of the presence of micro-metastases; followed by radiation therapy to the operative site. The patient received five cycles of chemotherapy consisting of vincristine, adriamycin, cyclophosphamide alternated with ifosphamide and etoposide. The patient’s left ventricular ejection fraction showed a decrease after five cycles of chemotherapy and the patient then went on to receive radiation treatment. She received a total radiation dose of 50 Grays to the whole breast and 60 Grays to the operative site. The patient tolerated the treatment well and is currently completely symptom free and clinically well one and half years after completion of her treatment.

## Discussion

Phyllodes tumours, despite being benign, have a tendency to recur after surgery. An originally histologically benign tumour may develop malignant features with recurrence [1]. The literature shows that in most cases more aggressive growth and enhanced malignancy is found on recurrence [2]. Recurrences may result from proliferative remnants of the primary tumour following local excision or they may be de-novo tumours induced by an extra-tumoural stromal hypercellularity resulting in a new benign cystosarcoma phyllodes [3]. They behave more like soft tissue sarcomas rather than tumours arising from the glandular tissue of the breast [4]. Little correlation exists between the biological behaviour and the histological appearance of cystosarcoma phyllodes [4], independent of the surgical procedure [1]. Metastases have been observed in 19% of malignant phyllodes tumour [3]. The interval between the diagnosis of the primary tumour and identification of metastases ranged from seven months to five years, whereas the interval between recurrent tumour and metastases was from 6 to 24 months or in some cases matastases were present simultaneously [3]. Very rarely, the malignant potential increases and results in the conversion to a sarcoma of the breast. Fibrosarcomas are amongst some of the most rare tumours of the breast. Any breast neoplasia that does not display characteristics of a fibroadenoma are designated stromal sarcoma of which fibrosarcomas are a small percentage. They may occur at any age, but are commonly seen in women between 40 and 50 years [5]. There are no characteristic features that clinically distinguish them from other breast tumours. In this case study, a review of the slides was done to confirm the diagnosis although the lack of immunohistochemistry is a potential drawback in this case. Metastases from fibrosarcoma breast are commonly seen in the lung but may occur in the brain, kidney and the bone; lymphatic spread is rare [5]. The prognosis for fibrosarcoma of the breast is poor and the role of various treatment modalities is not clearly defined due to rarity of disease [6]. Careful preoperative multi-disciplinary assessment is required before making the therapeutic decision [8]. Surgical treatment should consist of at least a simple mastectomy and all attempts should be made to achieve negative margins as this appears to be the major factor influencing survival in these patients [7]. Axillary lymph node dissection is not indicated [6]. Studies suggest that if negative surgical margins can be achieved, then breast sarcoma should be managed by conservative surgery with postoperative irradiation with a dose of 50 Gray to the whole breast, and at least 60 Gray to the tumour bed [8]. Tumour size may be more important prognostic factor than tumour grade [2]. As large tumour size and positive surgical margins incur a higher risk for local failure, radiotherapy is probably indicated in these selected cases and should be given within four months of surgery [6]. Radiation treatment alone after mastectomy may fail to prevent a recurrence from the original tumour, whether benign or malignant [1]. Chemotherapy can be used when chances of distant metastases are high. Distant metastases are developed in 3.2%, 11.1% and in 28.6% of patients with benign, borderline and malignant phyllodes tumours, respectively [9]. With histopathological conversion to a sarcoma, the chances of distant metastases increase. Established therapeutic principles and techniques used for both soft tissue sarcoma and breast cancer should be applied [6]. Chemo-radiation may have a role in large tumours where an aggressive approach is required as in any soft tissue sarcoma whereas surgery with or without radiotherapy remains the standard treatment [10].

## Conclusion

Phyllodes tumour of the breast has a tendency for recurrence after surgery with increased malignant potential. In rare instances, it may convert to fibrosarcoma of the breast. Such cases can be treated successfully with a combined modality treatment of surgery, chemotherapy and radiotherapy.
